# Molecular characterization of *Coxiella burnetii* in livestock species in Isiolo County, Kenya and the associated risks factors for seropositivity

**DOI:** 10.3389/fvets.2026.1797693

**Published:** 2026-05-04

**Authors:** Enock Kiprono, Hussein M. Abkallo, Richard Nyamota, Lynn J. Kirwa, Reuben Mwangi, Athman Mwatondo, Eugine Mukhaye, Mathew Muturi, Joel L. Bargul, James M. Akoko, Bernard Bett

**Affiliations:** 1 Health Program, International Livestock Research Institute (ILRI), Nairobi, Kenya; 2 Department of Biochemistry, Jomo Kenyatta University of Agriculture and Technology (JKUAT), Nairobi, Kenya; 3 KEMRI-Wellcome Trust Research Programme, Kilifi, Kenya; 4 Kenya Zoonotic Disease Unit, Ministry of Health and Ministry of Agriculture, Livestock and Fisheries, Nairobi, Kenya; 5 Department of Veterinary Medicine, Dahlem Research School of Biomedical Sciences (DRS), Berlin, Germany

**Keywords:** abortion, *Coxiella burnetii*, phylogenetics, Q fever, qPCR, zoonoses

## Abstract

**Background:**

*Coxiella burnetii* is the causative agent of Q fever, a zoonotic infection that poses serious threats to both animal and human health, particularly in the Global South. It infects livestock such as cattle, sheep and goats including wildlife and can lead to both agricultural and economic losses.

**Methods:**

A cross-sectional study was conducted to collect blood and serum samples from aborted livestock; 18 Cattle, 22 Sheep, and 72 goats from March 2022 to August 2023. The samples were screened for antibodies against *Coxiella burnetii* and the nucleic acids for *Coxiella, respectively.* Before the onset of our study, all the three livestock species; goats, cattle and sheep in Kinna ward were first sampled to determine the presence of *C. burnetii*. A total of 275 collected samples were screened for prior exposure of *C. burnetii* and its results were used as reference for this study thus referred to as baseline study.

**Results:**

A total of 387 samples were analyzed consisting of 112 from the cross-sectional study and 275 from the baseline study to determine the presence of anti *C. burnetii* antibodies. Our results showed that goats had higher seropositivity 49.65% (71/143, 95%CI: 41.97–57.75) followed by sheep 16.67% (15/90, 95%CI: 10.37–25.69) and cattle 3.25% (5/154, 95%CI: 1.40–7.37). Livestock species with a history of abortion had higher seropositivity of 33.89% (goats 57/62, sheep 4/62, cattle 1/62, 95%CI: 27.38–41.08) compared to non-aborting from the baseline study (goats 15/30, sheep 11/30, cattle 4/30, 95%CI: 10.34–19.93). Conditional logistic regression model identified abortion as a significant risk factor, with goats being 26.71 times more likely to abort than cattle, and sheep 3.59 times more likely than cattle. Among the 112 blood samples, 54 constituting 34 goats, 12 cattle and 8 sheep tested positive by qPCR and 16 of these were subjected to Sanger sequencing.

**Conclusion:**

Phylogenetic analysis, performed using the maximum likelihood approach, provided insights into the genetic diversity and circulation of *C. burnetii* strains. Findings from this study will support the development of intervention strategies such as vaccination or biosecurity improvements aimed at reducing abortion rates and economic losses in small ruminant and cattle production systems.

## Background

Q fever, caused by *Coxiella burnetii*, is a contagious zoonotic infectious disease of public health concern (1). According to WHO 2022 report (2), Q fever is a widespread zoonotic disease that significantly affects low-and-middle income countries, particularly in rural and farming communities where people frequently come in contact with infected livestock. In these regions, limited access to healthcare, diagnostic services and treatment often leads to underdiagnosis and underreporting of cases (3) In addition, public awareness remains low thus referred to as Neglected Tropical Disease (NTD). Q fever has been documented in over 59 countries worldwide, with the notable exceptions of New Zealand and Antarctica (4, 5). The disease was first identified in Montana, United States and Queensland, Australia highlighting its global reach and importance of monitoring its spread across different regions (6). Q fever seroprevalence has been reported to range from 13–24% in goats, 4–44% in cattle, 11–33% in sheep, and 1–32% in humans (7). Molecular detection studies in Africa indicate a prevalence of 9% in cattle, 16% in sheep, 23% in goats and 3% in humans across 24 out of 54 surveyed countries (8). In Kenya, most of the studies have focused on seroprevalence, estimating it at 28.2–57.1% in livestock population in pastoral communities, with limited data on molecular prevalence of the pathogen (9, 73).

Although *C. burnetii* infects a variety of animal species including wildlife, livestock such as goats, cattle, sheep are believed to be the primary source of Q fever infections in humans (10) due to close human-livestock interactions, especially in pastoral communities. *C. burnetii* transmission among livestock species can occur through tick vectors as ticks may harbor the pathogen and thus play a role in its transmission among domestic animals (11). The pathogen is shed through birth products, semen, milk, urine, feces and vaginal mucus (6, 12, 13). While Q fever has the potential to affect domestic animals, it is more prone to causing abortion in small ruminants (14). Furthermore, abortion and the subsequent discharge of fluids serve as a primary means of environmental transmission, increasing the risk of widespread infection among both animal and human population (15–17). Abortion in livestock is associated with a huge socioeconomic burden, which is felt directly through loss of herds. This results in heightened poverty levels in the global south especially in livestock-dependent livelihoods.

Limited access to sensitive diagnostic tools contributes to frequent misdiagnosis of Q fever. In humans, Q fever infections manifest itself as febrile illness, which is treated as presumptive malaria resulting in missed opportunities to accord, identify and treat other causes of the fever (18). Molecular diagnostics such as Quantitative real-time PCR (qPCR) has shown its sensitivity in identification of *C. burnetii* when used to target the IS*1111* insertion element, which is present in 20–30 copies (19–22). Despite the reliability of molecular techniques in the detection of *C. burnetii*, it is not frequently used due to limited expertise and its associated cost (23).

The unavailability of data on phylogeny of *C. burnetii* in Livestock in Kenya makes it difficult to understand its epidemiology. This study aims to determine seroprevalence and characterize the genetic diversity of *C. burnetii* in livestock from Isiolo, northern Kenya. Our findings will enhance knowledge of Q fever epidemiology and support the development of targeted interventions to prevent its emergence and re-emergence. To the best of our knowledge this is the first study investigating the genetic diversity of *C. burnetii* in livestock in Kenya.

## Materials and methods

### Ethical approval

Ethical clearance for this study was obtained from the International Livestock Research Ethics Committee; reference number ILRI-IREC2020-07.

### Study site

**Figure 1:**
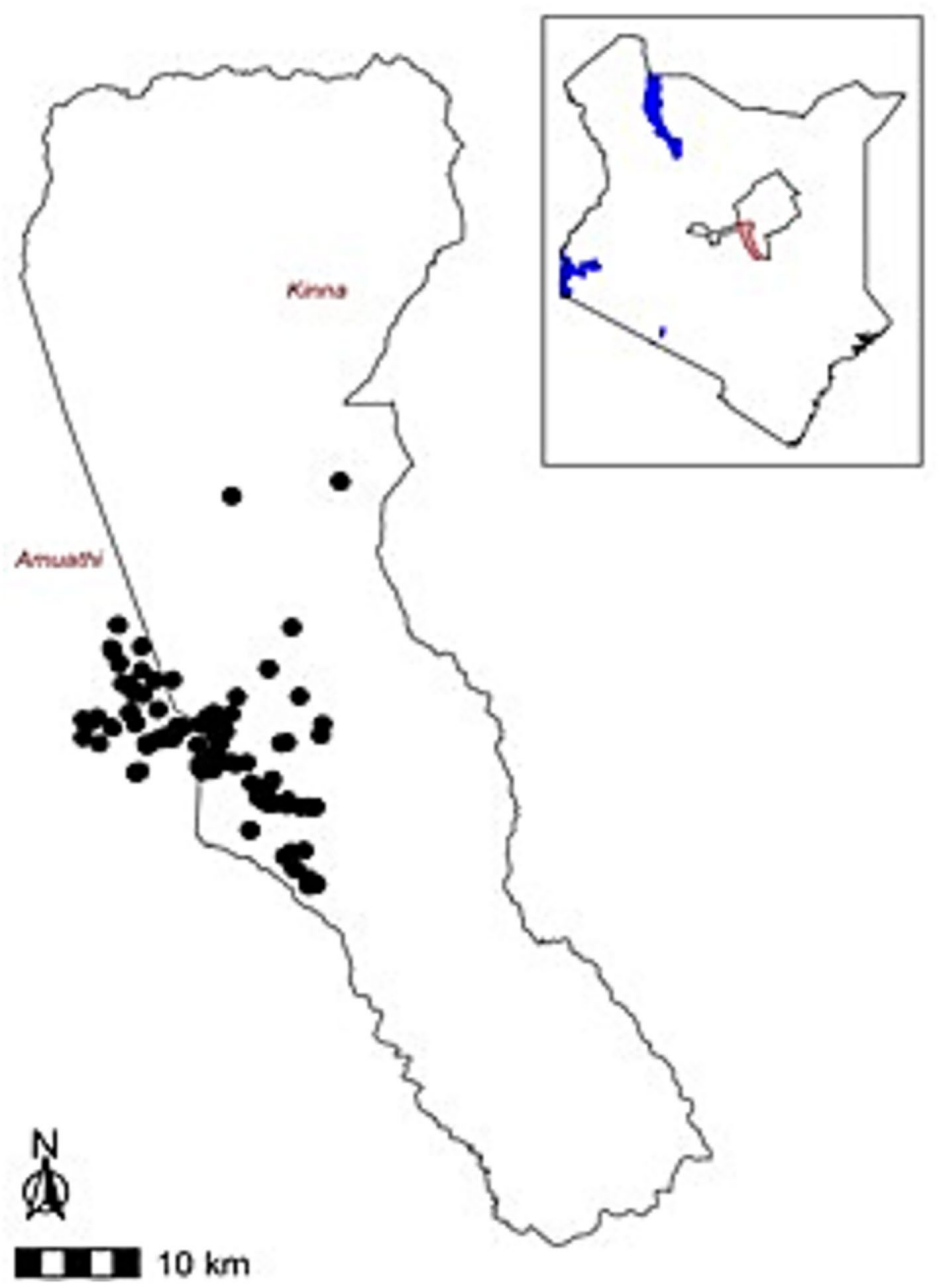
Map of Kenya showing the sampling points and sampling site Kinna ward, Garbatula sub-county within Isiolo County.

This study was conducted in Kinna, Garbatula sub-County, in northeastern part of Isiolo County, Kenya. The sampling points are illustrated in Figure 1, with some points falling outside Kinna ward as animals moved to neighboring wards in search for pasture. The area receives an average annual rainfall of 580 mm, ranging from 350 mm to 600 mm. Short rain occurs between November and December while long rains fall between March and May. The annual temperature ranges from 24 °C to 30 °C. The Borana tribe is the most predominant community in the area, with pastoral livestock production systems being their main source of income and livelihood. Three livestock species; goats, sheep and cattle are raised by over 80% of the population with goats and sheep having a higher population. This study site was chosen due to its proximity to Meru National Park, where wildlife and domestic livestock interact, and creating potential zoonotic transmission hotspots. Kinna also has a more stable animal population because of less migration and the area is also characterized by good accessibility.

### Study design and sample size estimation

A syndromic surveillance was conducted between May 2022 to August 2023 targeting animals presenting with suspected abortion related complications. The surveillance targeted goats, cattle and sheep. When abortion cases were reported, field veterinarians were alerted by the famers who then responded by visiting the affected households record the abortion event where blood and serum samples were collected alongside the metadata of the aborted livestock.

The sample size (*n*) used was determined using the equation below (24):


$$n=\frac{\left(1.92)p(1-p\right)}{d^{2}}$$

Where: *p* represents prevalence and *d* is the allowable error of 0.05.

A previous study conducted in a similar setting reported an average prevalence of *C. burnetii* in cattle, goats and sheep was 10.81% (25). Therefore, using the average prevalence of 10.81% reported in this published study and the aforementioned equation, the minimum sample size for serological analysis was estimated at 74. A total of 112 livestock species that experienced abortions during the study period constituted the total number of samples that were analyzed in this study. Blood samples were screened for *C. burnetii* infections using both serological and molecular assays. Before the onset of our study, all the three livestock species; goats, cattle and sheep in Kinna ward were first sampled to determine the presence of three pathogens including *C. burnetii.* This was done to establish the initial prevalence and distribution of the pathogens in livestock population. All samples collected were screen for prior exposure of *C. burnetii and* its results were used as reference for this study thus referred to as baseline study. Serological results of this cross-sectional study; aborted livestock were analyzed against the results of the baseline study; non aborting livestock.

To compare aborting and non-aborting livestock population, we used Epitool (26) to compute the minimum required sample size. Working with expected proportion of 0.05, assumed odds ratio of 4, confidence level 0.95 and the power of 0.8, the minimum sample size was 98. While a higher ration is preferred, our target comparison ration for aborted and non-aborted livestock was 1:4; however, we worked with a 1:2 ratio due to limited controls.

### Sample collection

Qualified veterinarians from ILRI, assisted by trained community field workers, collected blood samples while ensuring aseptic techniques. Cattle were led into a crush while goats and sheep were manually restrained by holding under the jaw while minimizing unnecessary pain or distress. Blood was drawn from the jugular vein into two 10 mL plain vacutainers (BD, Becton, Dickinson, Franklin Lake, United States) pre-labeled with unique barcodes. The red-top vacutainer was used to collect blood for serum extraction, while purple-top vacutainer was used to collect whole blood. Samples were stored at 4–8 °C in cool boxes with ice packs and transported to a field laboratory, where serum was extracted by centrifugation at 2,500 × g for 10 min. Both serum and EDTA blood samples were transferred to International Livestock Research Institute (ILRI) laboratories in Nairobi, Kenya, in a motorized freezer maintained at −20 °C for further analysis.

### Data collection

Structured questionnaires were administered by veterinarians using Open Data kit (ODK) to collect house-hold level data. The recorded information included animals with recent history of abortion, the number of previous cases of abortion or stillbirths, and incidences of delivery of weak calf births. A total of 112 livestock samples were collected, corresponding to recorded abortion cases. Households were randomly selected based on the occurrence of an abortion event.

### Serological assays

Serum samples were tested for antibodies against *Coxiella burnetii* antibodies using the ID Screen Q Fever Indirect Multi-Species Kit (ID VET, Montpellier, France) following the manufacturer ‘s instructions (27, 28). Briefly, 5 μL of the serum samples were diluted in 245 μL of the dilution buffer (1:50 dilutions) and loaded on a pre-coated plate. The plate was then incubated at 21 °C for 45 min and then washed three times with washing solution. Briefly, 100 μL of 1 × conjugate was added to each well and incubated at 21 °C for 30 min. It was washed three times before adding 100 μL of substrate to each well. It was then incubated for 15 min at 21 °C and thereafter 100 μL of stop solution was added to each well. Optical density measurements were recorded at a wavelength of 450 nm using a syn-energy BioTek microplate ELISA reader (Synergy, BioTek, Winooski, VT, United States).

### DNA extraction and detection of *Coxiella burnetii* using qPCR

Total DNA was extracted from 112 blood samples using Qiagen DNeasy blood extraction kit (Qiagen, Hilden, Germany) following the manufacturer‘s instructions. The extracted DNA was subject to Real Time PCR (qPCR) assay to detect *C. burnetii* targeting the *IS1111* gene using primers; Cox *IS1111* F5′CATCACATTGCCGCGTTTAC 3′ and Cox *IS1111* R 5′GGTTGGTCCCTCGACAACAT3′ and probe FAM 5′AATCCCCAACAACACCTCCTTATTCCCAC 3′ TAMRA (29). The 20 μL final reaction volume comprised of 10 μL Luna Universal Probe qPCR Master Mix (New England Biolabs, Ipswich), 0.8 μL of each 10 μM forward and reverse primers and 0.4 μL of Cox IS111 probe. Thermocycling conditions were 95 °C for 1 min, 95 °C for 15 s for 45 cycles and 60 °C for 30s. To validate the assay, limit of detection (LOD) was ascertained by generating a standard curve of positive control dilutions (from 18,000 copies/μL to 1 copy/μL). The limit of detection was established at Ct value of 35.113, meaning samples with Ct < 35.113 were considered positive. The qPCR reactions were performed using a Quant Studio 5 real time PCR systems thermocycler (Thermo Fischer scientific, United states).

Conventional nested-PCR amplification of *Coxiella burnetii IS1111* gene was done on the qPCR positive samples using Cox F1 5-TATGTATCCACCGTAGCCAGTC-3′ and Cox R15-CCCAACAACAACCTCCTTATTC-3′primers for the primary reaction generating a 685 bp fragment (30). The primary PCR products were used as the PCR template for the secondary reaction using Cox F2 5-GAGCGA ACCATTGGTATCG-3 and Cox R2 5-CTTTAACAGCGCTTGAACGT-3′ primers for the secondary reaction that generated 201 bp fragment (31). The reaction was set up in a 25 μL reaction volume consisting of 12.5 μL of Q5 high fidelity 2 × master mix (New England Biolabs), 1.25 μL of both forward and reverse primer, 8 μL of Nuclease free PCR water and 2 μL of the template DNA. Amplification conditions: 98 °C initial denaturation for 30s followed by 35 cycles of 98 °C for 10 s, 54 °C for 30 s, 72 °C for 20 s and final extension at 72 °C for 2 min. Gel electrophoresis was done on nested PCR amplicons to visualize the amplified fragment. Bi-directional Sanger sequencing was done on the purified secondary PCR products that generated the expected band size. Raw forward and reverse sequences for each sample were assembled using Seq Trace version 0.9.0 to generate consensus sequence (32). Sequences obtained from this study were deposited in GenBank and have been assigned accession numbers; OR684935 to OR864950.

### Phylogenetic analysis

The sequences form this study were used to query the NCBI database for similar *C. burnetii* sequences using BLAST. The identification of similar sequences was conducted using a BLASTn (BLAST^1^) accessed on January 8th 2025 (33). The top hits were retrieved based on the percentage identity and the *E*-value. Sequences that had a higher percentage identity and lower *E*-value were preferred as it indicated a more reliable and significant alignment between the query and target sequence (34). The retrieved sequence and those from this study were used to generate multiple sequence alignment MSA using MUSCLE (35) embedded in MEGA version (35). The MSA was used to determine the optimal maximum likelihood phylogenetic model based on Bayesian Information Criterion (BIC) (36). The robustness of the phylogenetic tree was ascertained by performing bootstrap analysis of 1,000 replicates. Phylogenetic tree was visualized using fig tree (37). Another phylogenetic tree was generated using similar approach to categorize our isolates into five IS1111 genomic groups. Reference strains for group I to group V (38) were retrieved from NCBI database, performed multiple sequence alignment using MUSCLE to generate evolutionary relationships between our isolates and the reference strains (39).

### Statistical analysis

All analyses were done using the R statistical package version 4.3.0 (40) whereby Chi-square (χ^2^) test was used to assess the relationship between categorical variables for independence. Seropositivity was first determined from all the samples within a confidence interval of 95%. For case control analysis, three livestock species; goats, cattle and sheep were analyzed where cases refer to the number of abortions recorded during the study period and the controls were drawn from the initial screening of *C. burnetii* antibodies in the three livestock species before the onset of the project. Conditional logistic regression model was used to fit the cases and control using clogit model (41) in R statistical package. Odds ratios were calculated to determine the magnitude of the risk factors.

## Results

A total of 387 samples were analyzed consisting of 112 from the cross-sectional study and 275 from the baseline study to determine the presence of anti *C. burnetii* antibodies. Considering the livestock species, the highest seropositivity was reported in goats 49.65% (71/143, 95%CI: 41.97–57.75) followed by sheep with a seropositivity of 16.67% (15/90, 95%CI: 10.37–25.69) while cattle had a seropositivity of 3.25% (5/154, 95%CI: 1.40–7.37). Livestock species with a history of abortion had a seropositivity of 33.89% (goats 57/62, sheep 4/62, cattle 1/62, 95%CI: 27.38–41.08) compared to 14.39% (goats 15/30, sheep 11/30, cattle 4/30, 95%CI: 10.34–19.93) in the non-aborting population. The values for both categories; livestock species and abortion were found to be significant as shown in Table 1.

**Table 1:** Seropositivity estimates of *Coxiella burnetii* with 95% confidence interval in three livestock species.

Variable	Category	Positive	Negative	% seropositivity	95% CI	*P*-value
Livestock spp.	Cattle	5	149	3.25	1.40–7.37	<0.001
	Sheep	15	75	16.67	10.37–25.69	
	Goats	71	72	49.65	41.97–57.75	
Abortion	No	30	177	14.49	10.34–19.93	<0.001
	Yes	61	119	33.89	27.38–41.08	

Comparison between aborting and non-aborting livestock species was done as shown in Table 2. Abortion was identified as a potential risk factor, with an odds ratio (OD) of 1.14 (95%CI; 0.53–2.28). However, the association was not statistically significant (*p* = 0.735). Among the three livestock species sampled, goats had 26.71 times higher odds of experiencing abortions compared to cattle (95%CI; 8.49–84.10, *p* = 0.001) indicating a statistically significant association. Sheep were 3.59 times more likely to abort than cattle 95%CI (0.95–13.63), but this association was not statistically significant (*p* = 0.06).

**Table 2:** Conditional logistic regression analysis for aborting and non-aborting livestock population.

Descriptives	Control	Cases	Coef	95%CI	*P-*value
No abortion	387		1 (ref)		
Abortion	387	180	1.14	0.53–2.48	0.735
Cattle	154	27	1 (ref)		
Sheep	90	45	3.59	0.95–13.63	0.06
Goats	143	108	26.71	8.49–84.10	<0.001

### Molecular detection and phylogenetic analysis

**Figure 2:**
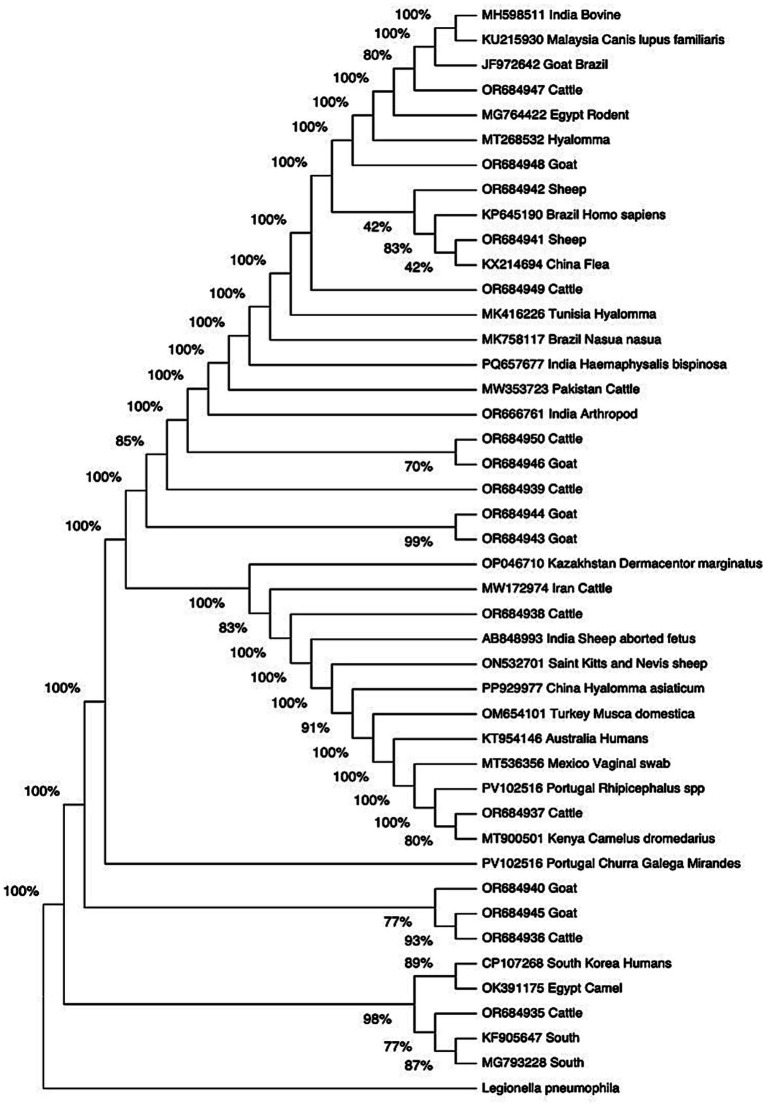
Phylogenetic tree showing evolutionary relationship of our isolates and sequences retrieved from NCBI. This tree was constructed using maximum likelihood approach, Kimura 2 model based on COX IS 1111 gene. The numbers at the node represent the support of each node. These nodes labels range from 0 to 100% with 100% representing maximal support.

Out of 112 extracted DNA samples, *Coxiella burnetii* DNA was detected in 54/112 (48.2%) by qPCR with the highest detection in goats (30) followed by cattle (13) and finally sheep (11). Among the qPCR-positive samples, 16 samples were successfully amplified using nested PCR. BLAST analysis showed sequence similarity ranging from 86.52 to 100% with existing *Coxiella burnetii* sequences in GenBank, with the Expect value (*E*-value) of zero and below indicating that the match was significant. A phylogenetic tree was constructed using both Isiolo and global isolates (shown in Figure 2). Kimura-2 model was used because it had the lowest BIC (42) and phylogenetic reconstruction was done using Maximum Likelihood approach. The analysis revealed that our isolates were distributed across the entire tree, falling into different clades. Three major clades were identified, with members of the same clade exhibiting varying branch lengths. Notably, two isolates from same species (goats) clustered together with the rest of the isolates clustering with other isolates from different hosts in different geographical location.

## Discussion

Livestock species: goats, cattle and sheep could serve as the primary reservoirs for *C. burnetii*. Since our test was focused on detecting IgG antibodies, presence of *C. burnetii* antibodies in blood indicates the possibility of prior exposure to the pathogen (43). Frequent contact between infected and susceptible livestock at the grazing field could enhance its transmission as the pathogen is extremely infectious (39, 44). This risk is exacerbated in livestock managed under extensive feeding management systems as they are more prone to acquiring infections compared to those under intensive systems, likely due to increased movements, herd interactions and exposure to contaminated water sources (45, 46). Our study recorded a lower *C. burnetii* seropositivity in cattle compared to goats and sheep. A study conducted in 2023 showed that herd size was directly proportional to the seropositive of *C. burnetii* in cattle (47). Shedding patterns play a role in the prevalence of *C. burnetii* with cattle mainly shedding it through milk and to a lesser extent through Vaginal mucus (11, 48). Conversely, goats and sheep have multiple shedding routes; Vaginal mucus, feces and milk which enhance its prevalence and reinforce its transmission to the environment (47, 48).

Our study found higher seropositivity in goats than in sheep and cattle, consistent with previous study in almost similar settings (9). This is in line with previous study done in Kenya that categorized goats and sheep to have a higher seroprevalence as compared to cattle (49). Since *C. burnetii* contaminates environment, especially soil and dust, studies have shown that animals that graze or browse closer to the ground are more likely to come in contact with the pathogen (50). However, this was not the case for this study as we found out higher seropositivity in goats than sheep. Goats are primarily browsers while sheep are grazers feeding on low vegetation thus often in contact with the ground. Goats are more susceptible to infections by *C. burnetii* thus high prevalence than sheep (12). In addition, goats tend to shed more *C. burnetii* through vaginal mucus and feces and longer duration than sheep thus increasing environmental contamination and transmission risks within goats’ herds (51) (52, 53). According to a study conducted in 2013, goats may have intense exposure of abortifacient pathogens at the flock level (54). Abortion history plays a role in the presence of *C. burnetii* antibodies in goats. Studies have shown that goats with previous history of abortion are likely to be seropositive (48). While this study on *C. burnetii* seropositivity in goats cannot directly link the recorded abortion cases, we can hypothesize that the seronegative cases among aborted goats were caused by other abortifacient pathogens.

Our conditional logistic regression analysis indicated a higher frequency of abortion among the sampled three livestock. While *C. burnetii* was the focus of this study, some abortion cases may be attributed to other non-infections agents such as selenium deficiency (55, 56). Non-infectious agents were not investigated in this study. However, from the literature, these agents contribute approximately 10% of the abortion events (57). On the other hand, in most cases, presence of broad spectrum of infectious abortifacient pathogens such as *Chlamydia abortus, Brucella* spp., *Leptospira* spp. and viral agents such as *Rift Valley Fever virus* increases the chances of abortion to happen (57, 58). Other factors that have been documented to cause abortion in livestock include genetic factors, toxic chemicals, nutritional and metabolic problems (58). An experimental study has shown that *C. burnetii infection* does not always cause abortion, though it may occur under infection extreme conditions (29).

Our study identified goats as the most affected by abortion, reaffirming findings that cattle are less susceptible to abortions caused by *C. burnetii* (59). Some studies have documented abortion rates in goats to be about 90% implying that this livestock species is prone to infections and abortion (51, 58). Goats are particularly susceptible to *C. burnetii* during pregnancy (29) due to weak immune responses, which may explain why *C. burnetii* is responsible for abortion in goats in this study. A study done in 2019 depicted the seroprevalence of *C. burnetii* was 50% in the population of sheep aborted implying that the pathogen is circulating in the population and it could be the source of infection to other livestock within the flock (12, 60). Studies in aborted sheep showed the presence of *C. burnetii* based on serological evidence (61) although some studies have described the presence of *C. burnetii* in sheep as complicated because of the excretion of the abortifacient pathogen by healthy sheep (12). *C. burnetii* is known to cause abortion in sheep, especially in later stages of pregnancy when the pathogen is shed in large quantities (62). However, it is not the only pathogen responsible for abortion in small ruminants. Research indicates that abortion in sheep and goats is often caused by multiple factors including other pathogenic bacteria such as *Toxoplasma gondii, Campylobacter species, chlamydia abortus* with many cases involving coinfections rather than *C. burnetii* alone (58). In some cases, *C. burnetii* can be found in livestock species without any symptoms thus suggesting that infections do not always result in abortion (62).

With the use of molecular technique, qPCR, we detected *C. burnetii* in 54 of 112 (48.21%) nucleic acids extracted from whole blood consisting (34 goats-62.9%, 12 cattle-22.2%, and 8 sheep-14.81%). This indicates that this could be one of the pathogens linked to aborted as the other pathogens known to cause abortion were not investigated in this study. Similar studies done on molecular detection of *C. burnetii* on goats, cattle and sheep have demonstrated higher molecular prevalence in goats than sheep and cattle (31, 39, 63). Detection of this pathogen indicates recent infection implying that more goats were recently infected as compared to sheep and cattle. While no similar study has been conducted in Kenya, a study in Tanzania detected *C. burnetii* using the Cox IS*1111* gene, a well-established rapid and reliable molecular diagnostic marker (22). PCR detection using Cox IS*1111* gene has proven its sensitivity and specificity for detection of *C. burnetii* (28, 64). A study done in 2024 recorded a higher molecular prevalence in sheep (82%), goats (81%) and cattle (44%) (65). This could point out higher bacterial load or infection frequencies in sheep than goats and cattle. In most cases especially in pastoral communities, sheep tend to be kept in a larger flock where animals stay close together. This therefore increases the chances or risks for transmission through close contact with infected urine, feces and even birth products that contaminate the environment. In terms of excretion patterns, goats shed more *C. burnetii* through vaginal mucus with higher DNA quantities detected in environmental samples as compared to sheep (66). This could explain why low molecular prevalence in goats is recorded on blood samples than in environmental samples. Our study recorded a higher molecular prevalence of *C. burnetii* in cattle than sheep. This is in line with a study done in 2021 that recorded a higher prevalence on cattle aborted samples (21.7%) as compared to goats and sheep (16). In most cases, cattle tend to excrete *C. burnetii* in lower amounts as compared to goats and sheep (67). In terms of shedding routes and frequencies, cattle exhibit less intense and less prolonged bacteria shedding (67) and have a lower level of environmental contamination as compared to goats and cattle (66).

The phylogenetic tree generated from our data illustrates close evolutionary relationships between global and Isiolo isolates giving insights on the nature of pathogenic *C. burnetii* circulating in different regions globally. This is in line with finding from Das et al. (64) that showed similarities between isolates from India and Isolates from the global isolates based on the Cox *IS1111* gene. As per our findings, close evolutionary relationship was evident in all the isolated sequences with all the sequences falling into different clades sharing a common ancestor. The difference in members of the same clade could be pointed out by the branch length which signifies the level of evolutionary divergence from member of the same clade. This was evident in Figure 2 whereby there were variations in branch length. Based on our finding, the isolates from Isiolo county were distributed in different clades indicating their genetic relatedness with already existing sequences which is consistent with other finding done in 2019 (68) that found out limited evolution of *C. burnetii*. Interestingly, few of our isolates from the same sources were clustered together; OR684944 and OR684943 both isolated from goats while OR684950 isolated from cattle and OR684946 from sheep were clustered together. This depicts close evolutionary relationship sharing common ancestry. Studies have shown that different content in the insertion sequence is responsible for disparity in evolutionary change of *C. burnetii* (69, 70). This occurs as a result of genetic changes such as deletion occurring in various divisions of *IS1111* insertion element leading to the emergence of diverse strains (38). Therefore, we hypothesized that the emergence of diverse strains emanated from genetic changes occurring in *Cox IS1111* insertion element contributing to development of more divergent *C. burnetii* strains. Additionally, variations in livestock species and regional factors likely influence *C. burnetii* strain distribution.

**Figure 3:**
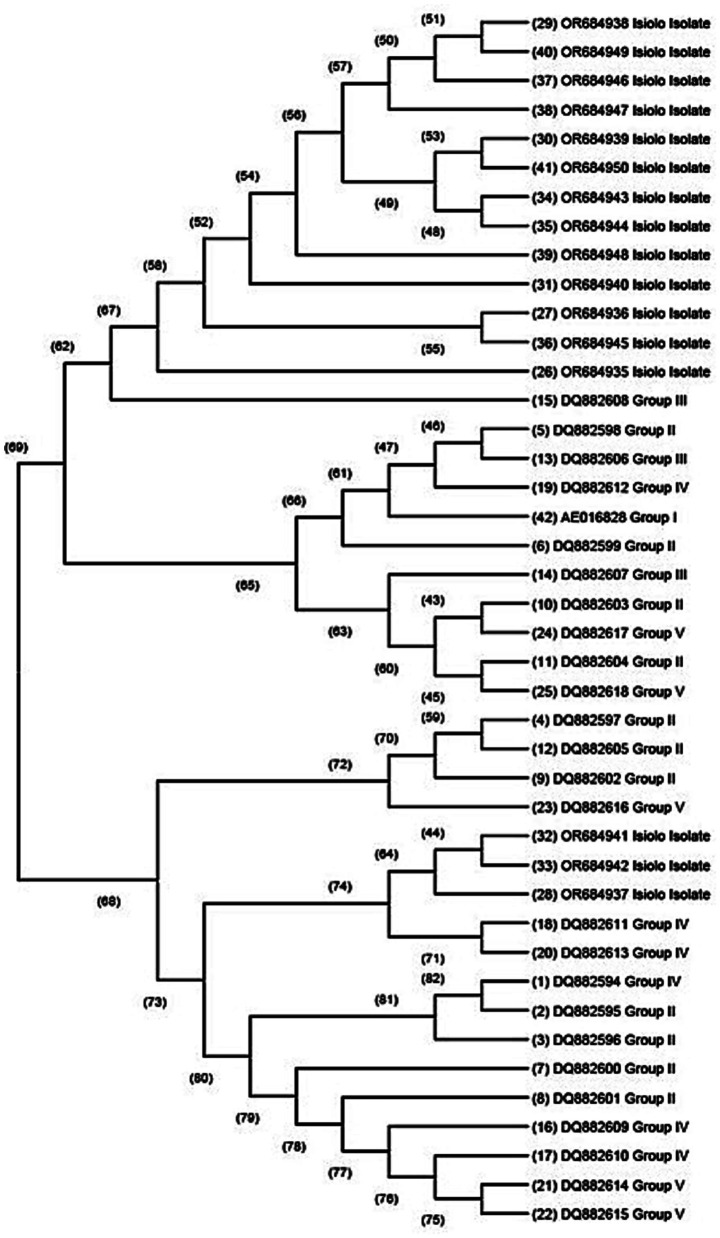
Phylogenetic tree showing evolutionary relationship of our isolates and *C. burnetii* genotypic groups I, II, III, IV, and V. This tree was constructed using maximum likelihood approach, Kimura 2 model based on COX IS 1111 gene. The numbers at the node represent the support of each node. These nodes labels range from 0 to 100% with 100% representing maximal support.

Our isolates were placed into five genomic groups using IS1111 algorithm (39) to depict how *C. burnetii* sequences isolated from livestock in Isiolo clustered with reference sequences for acute and chronic diseases. Findings showed that majority of our isolates were evolutionary related to DQ882608 reference sequence for group III while three of our sequences showed close relationship with DQ882611 and DQ882613 belonging to group IV as shown in Figure 3. The distinction between the genomic groups demonstrated clinical and epidemiological implications. Acute associated groups I, II, III have been linked to explosive outbreaks in ruminants characterized by late-term abortions, still births and extensive environmental contamination with *C. burnetii* (39, 71). In Livestock populations, presence of acute strains results in abortion storms that affects a high percentage of pregnant livestock with subsequent massive shedding of *Burnetii* in placental tissues and birth fluids (72). Conversely, chronic associated groups IV and V present an epidemiological pattern. In veterinary setting, chronic group isolates have been associated with reproductive losses, persistent herd infection, long term shedding cycles that are more challenging to be eliminated than acute outbreaks (72).

## Conclusion and recommendations

Although pathogenicity of *C. burnetii* was not assessed, this study showed genetic similarity between isolated sequence and other known pathogenic *C. burnetii.* From our findings, it is quite evident that *C. burnetii* is one of the abortifacient pathogens infecting livestock in our study area given that the pathogen was detected in aborted livestock population, although we are not ruling out co-infections with other abortifacient pathogens. Serological findings from this study provided evidence of prior exposure to infectious pathogens in livestock population which could be linked to reproductive losses thus underscoring the need for integrated disease surveillance and control programs.

Therefore, future studies could be focused on metagenomic analysis to determine other pathogens that could be linked to abortion in livestock species. In addition, it is worth screening the samples used in this study for other pathogens*, Toxoplasma gondii*, *Rift Valley Fever Virus*, *Brucella* and other pathogens that are known to cause abortion in livestock species. There is a need to carry out community sensitization, particularly among pastoralists, on proper handling and disposal of aborted materials. This will be of great essence in reducing and curtailing the spread of infection to humans. *Coxiella burnetii* circulating in Isiolo is of public health concern and therefore managing Q fever in both animals and humans is crucial. Furthermore, zoonotic control methods and continuous monitoring programs should be upscaled.

## Data Availability

The datasets presented in this study can be found in online repositories. The names of the repository/repositories and accession number(s) can be found below: https://www.ncbi.nlm.nih.gov/, OR684935-OR864950.

## Footnotes

1
http://www.ncbi.nlm.nih.gov/BLAST/
